# Supplementary material for: Two-Component System Sensor Kinases from Asgardian Archaea May Be Witnesses to Eukaryotic Cell Evolution
Source: Molecules. 2023 Jun 28;28(13):5042. doi: 10.3390/molecules28135042 (PMC10343646; doi:10.3390/molecules28135042)

Supplementary Figure 1

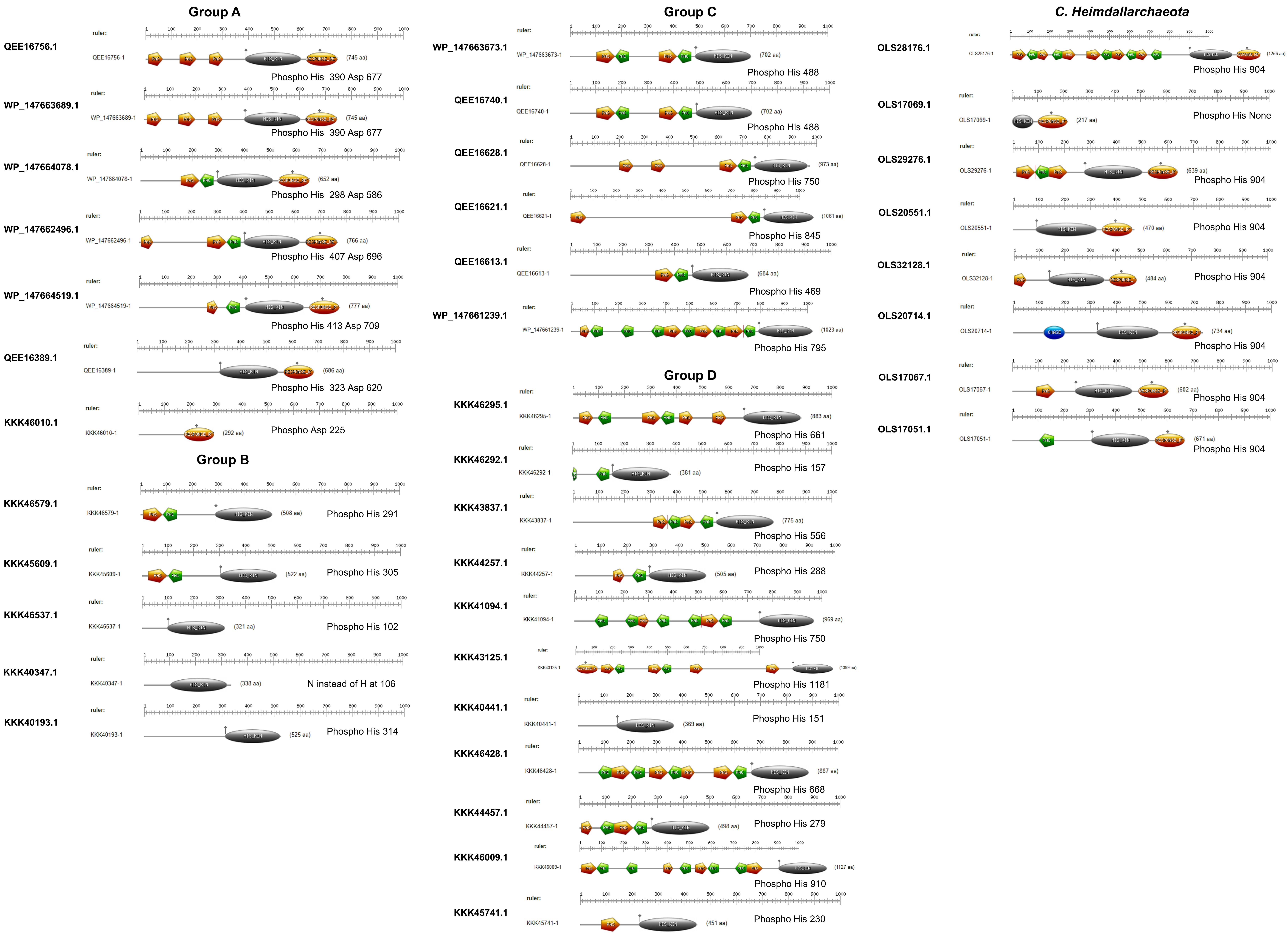

Supplementary Figure 2

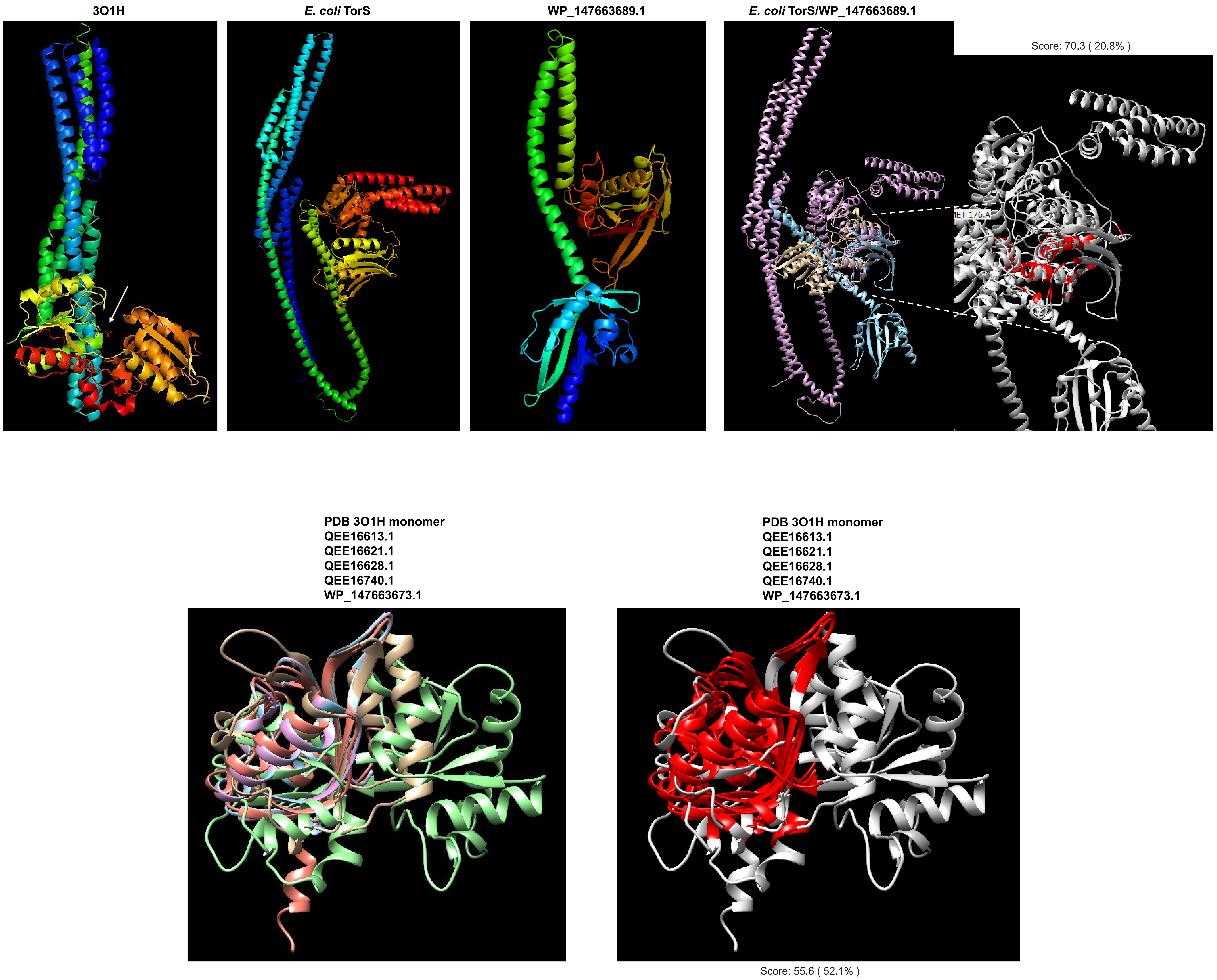

Supplementary Figure 3

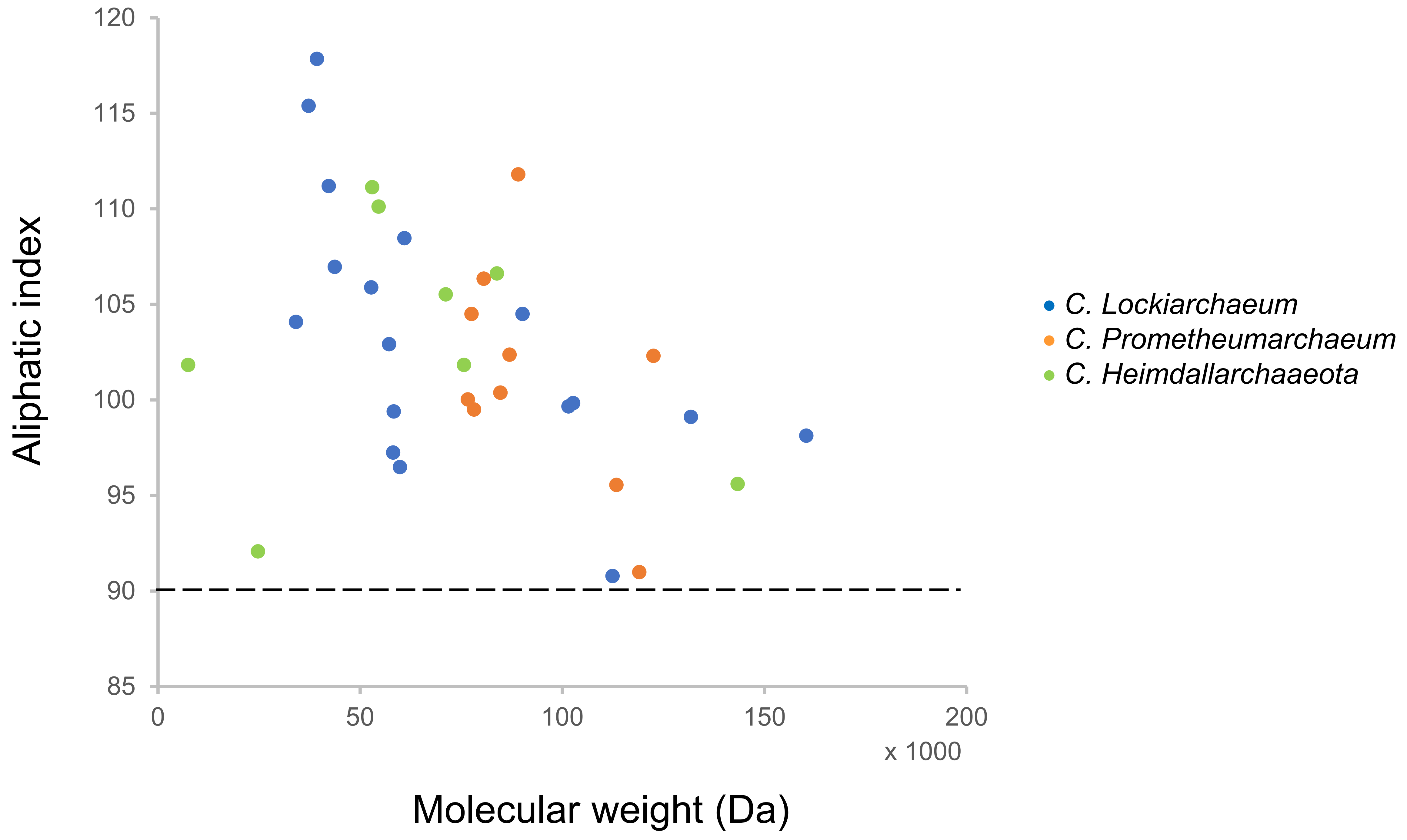

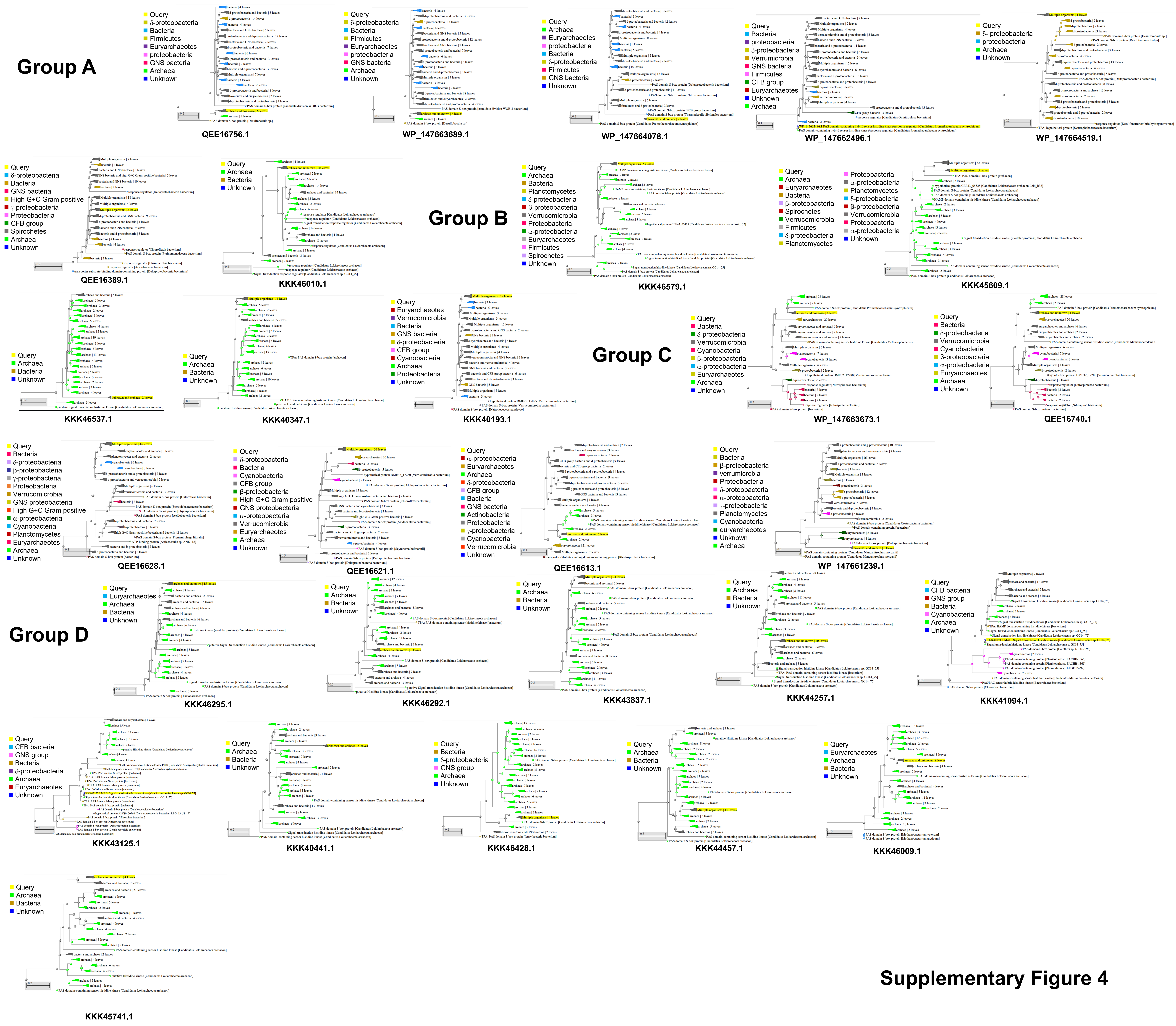

Supplementary Figure 4

Supplementary Figure 5

C. Heimdallarchaeota

OLS28176.1

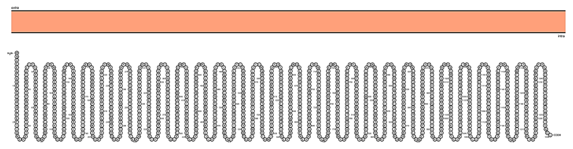

MW= 143408.00
pI= 5.45
Aliphatic Index= 95.60

OLS17069.1

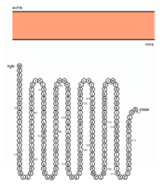

MW= 24789.13
pI= 4.61
Aliphatic Index= 92.07

OLS29276.1

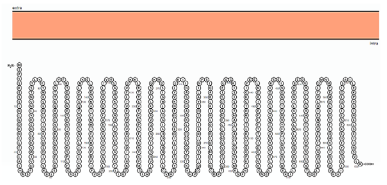

MW= 71195.58
pI= 4.81
Aliphatic Index= 105.52

OLS20551.1

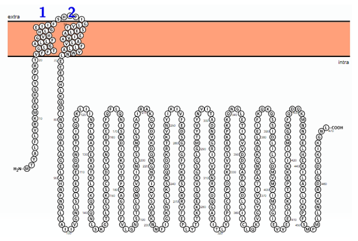

MW= 53006.90
pI= 7.07
Aliphatic Index= 111.13

OLS32128.1

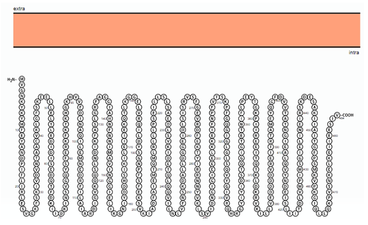

MW= 54598.91
pI= 4.96
Aliphatic Index= 110.12

OLS20714.1

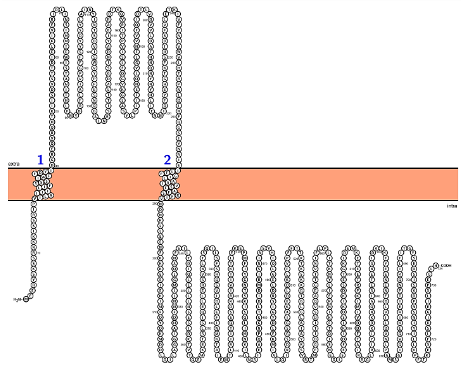

MW= 83863.09
pI= 5.00
Aliphatic Index= 106.62

OLS17067.1

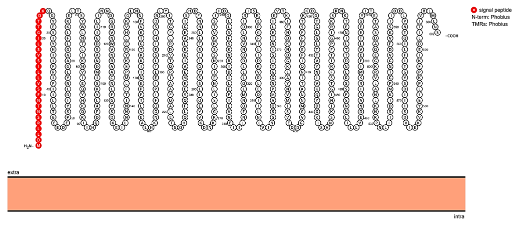

MW= 67917.14
pI= 5.33
Aliphatic Index= 113.97

OLS17051.1

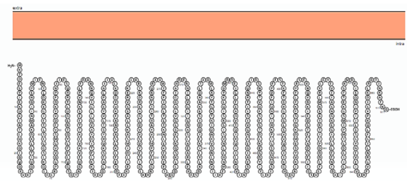

MW= 75716.97
pI= 5.21
Aliphatic Index= 101.83

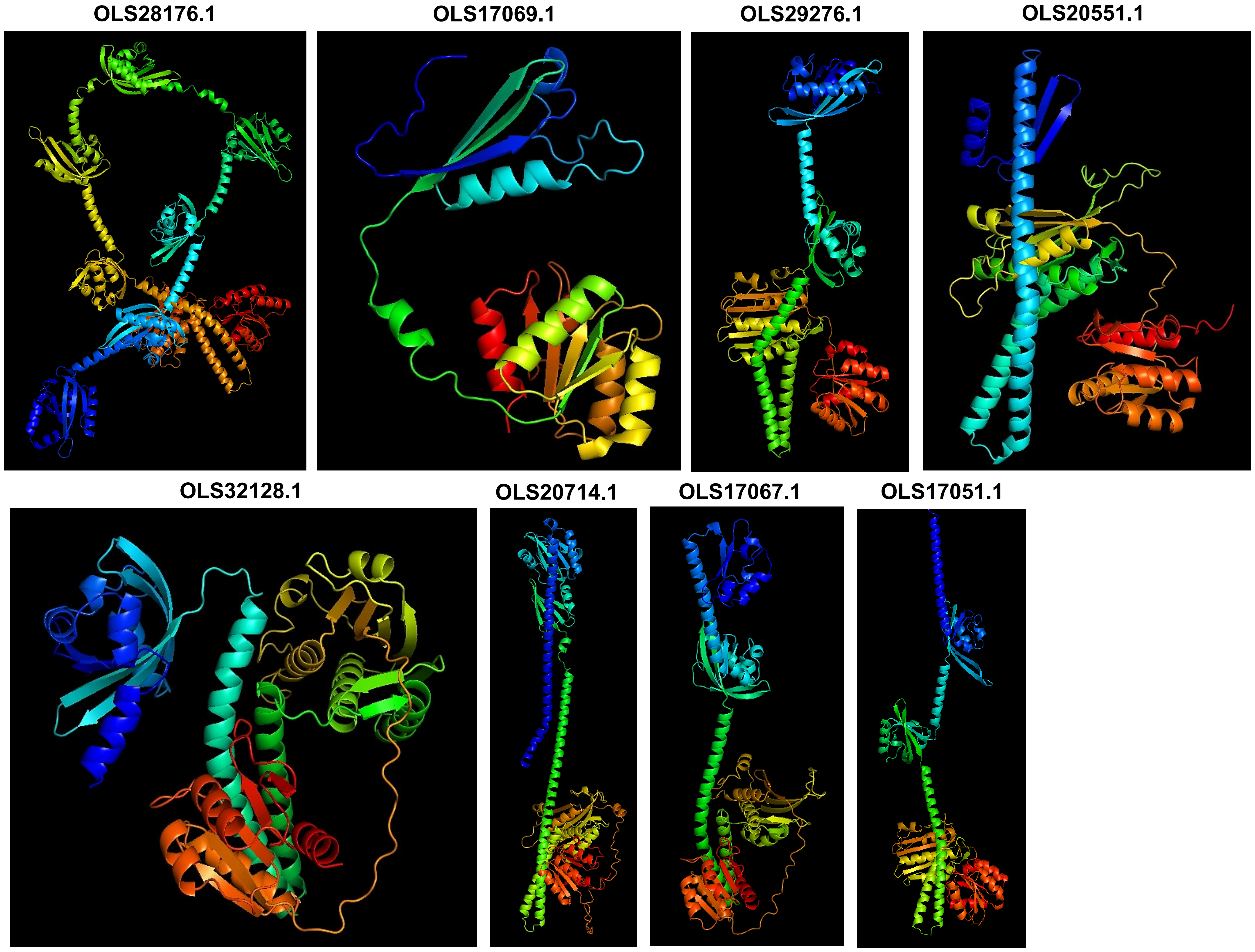

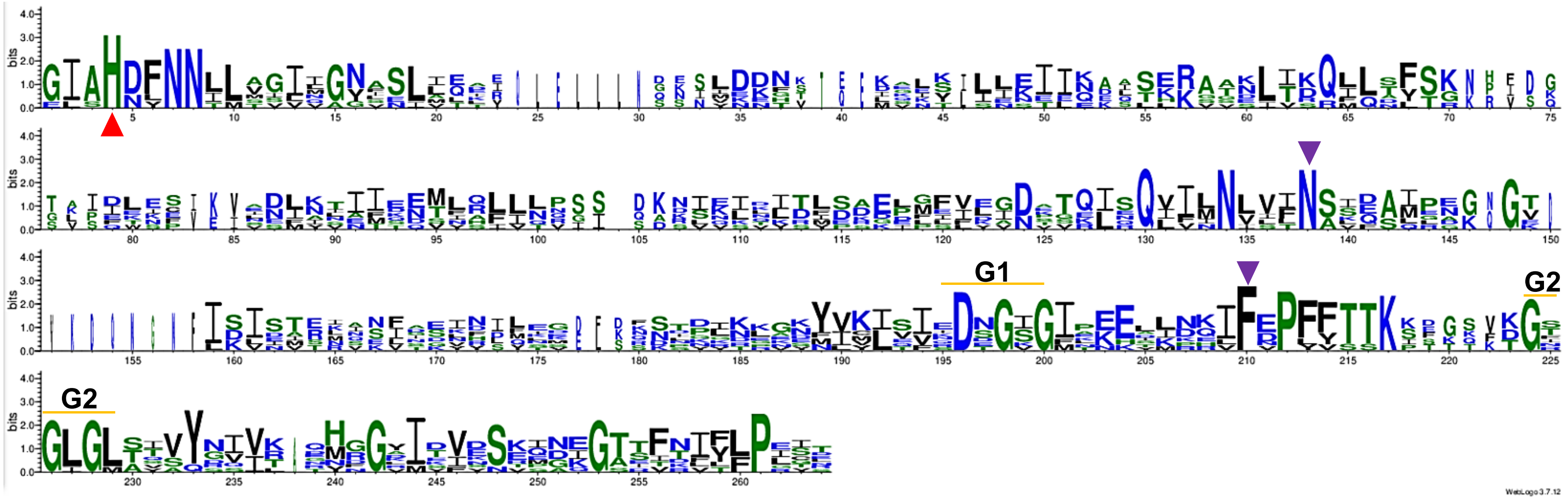

Supplementary Figure 6

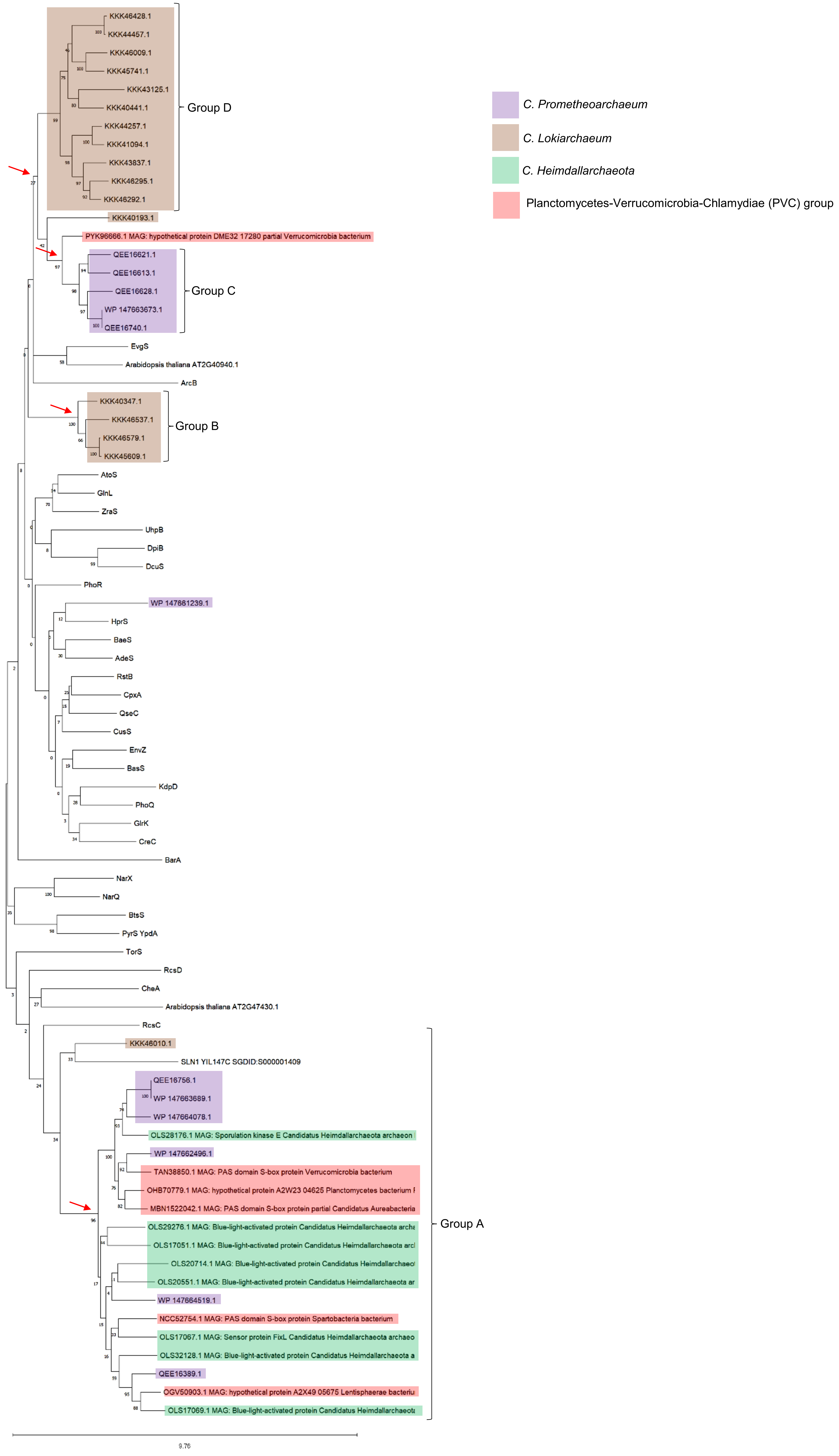

Supplement: Supplementary file 1 [file molecules-28-05042-s001.zip › Supplementary Figures Final.pdf]
