# Supplementary material for: Two-Component System Sensor Kinases from Asgardian Archaea May Be Witnesses to Eukaryotic Cell Evolution
Source: Molecules. 2023 Jun 28;28(13):5042. doi: 10.3390/molecules28135042 (PMC10343646; doi:10.3390/molecules28135042)
Supplement: Supplementary file 1 [file molecules-28-05042-s001.zip › Supplementary Figures Legends.docx]

**Supplementary Figure 1**. Domains found in TCS sensor kinases from the Asgardian archaea analysed in this work. Scan Prosite results are indicated, and the position of the modified residue on each protein is indicated.

**Supplementary Figure 2**. The TorS sensor domain near the catalytic domain is partially conserved in Asgardian bacteria. Top, the crystal structure of the TorS sensor domain (3O1H) and AlphaFold2 model of the full-length protein from *E. coli* and the WP_147663689.1 protein with a full-length domain. Comparison between these domains showed structural and sequence conservation when compared. The white arrow indicates the location of TMAO in 3O1H. Models of the remaining kinases having a TorS domain were individually modelled with AlphaFold2 and compared with the 3O1H structure (light green). In all instances, only part of the domain shows high homology, suggesting that these domains are partially present in these kinases.

**Supplementary Figure 3**. The aliphatic index of sensor kinases suggests that some proteins are less thermostable. Comparison between the molecular weight of each protein against their estimated aliphatic index using ProtParam56. The colour of each dataset is indicated. The dashed line indicates the limit for thermostability according to Ikai, 198044.

**Supplementary Figure 4**. Taxonomic distribution of each *C. Prometheoarchaeum* and *C. Lokiarchaeum* kinase. Blast Tree View is shown for the indicated kinase using fast minimum evolution and sorted by distance to identify the taxonomic group of each kinase. The legend contains the same colour scheme for each group as in the Blast Tree View output.

**Supplementary Figure 5**. Summary of *C. Heimdallarchaeota* kinase features. Here, the topology, overall protein model, and catalytic domain analysis are shown in Figure 2. The OLS170617.1 red region indicates a predicted secretion signal sequence.

**Supplementary Figure 6**. Phylogenetic reconstruction, including the top homologous sensor kinases from PVC bacteria using Blastp and all sequences shown in Figure 1 Panel B. Groups are indicated according to Figure 1 Panel B.

**Supplementary File 1**. Individual sequences for catalytic, phosphotransfer, and PAS domains in Asgardian kinases. The file is provided as a Microsoft PowerPoint modifiable file to allow retrieving sequences of interest.
