# Supplementary material for: Two-Component System Sensor Kinases from Asgardian Archaea May Be Witnesses to Eukaryotic Cell Evolution
Source: Molecules. 2023 Jun 28;28(13):5042. doi: 10.3390/molecules28135042 (PMC10343646; doi:10.3390/molecules28135042)
Supplement: Supplementary file 1 [file molecules-28-05042-s001.zip › Supplementary Information File S1.pptx]

## Slide 1
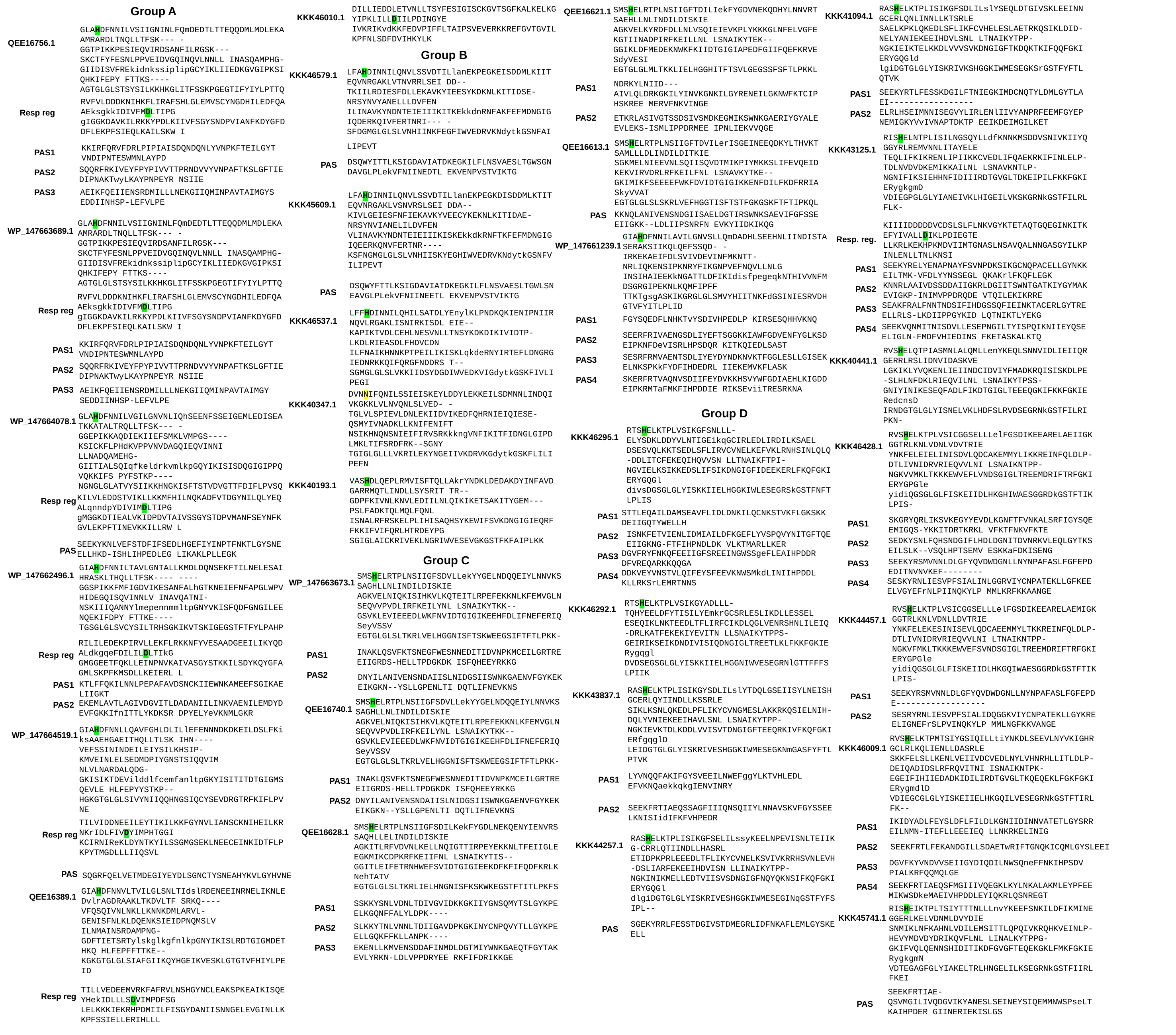

RASHELKTPLISIKGFSDLILslYSEQLDTGIVSKLEEINNGCERLQNLINNLLKTSRLE SAELKPKLQKEDLSFLIKFCVHELESLAETRKQSIKLDID-NELYANIEKEEIHDVLSNL LTNAIKYTPP-NGKIEIKTELKKDLVVVSVKDNGIGFTKDQKTKIFQQFGKIERYGQGld lgiDGTGLGLYISKRIVKSHGGKIWMESEGKSrGSTFYFTLQTVK
DILLIEDDLETVNLLTSYFESIGISCKGVTSGFKALKELKGYIPKLILLDIILPDINGYE IVKRIKvdKKFEDVPIFFLTAIPSVEVERKKREFGVTGVILKPFNLSDFDVIHKYLK
Group A
SMSHELRTPLNSIIGFTDILIekFYGDVNEKQDHYLNNVRTSAEHLLNLINDILDISKIE AGKVELKYRDFDLLNLVSQIEIEVKPLYKKKGLNFELVGFEKGTIINADPIRFKEILLNL LSNAIKYTEK--GGIKLDFMEDEKNWKFKIIDTGIGIAPEDFGIIFQEFKRVESdyVESI EGTGLGLMLTKKLIELHGGHITFTSVLGEGSSFSFTLPKKL
QEE16621.1
KKK41094.1
KKK46010.1
GLAHDFNNILVSIIGNINLFQmDEDTLTTEQQDMLMDLEKAAMRARDLTNQLLTFSK--- -GGTPIKKPESIEQVIRDSANFILRGSK---SKCTFYFESNLPPVEIDVGQINQVLNNLL INASQAMPHG-GIIDISVFREkidnkssiplipGCYIKLIIEDKGVGIPKSIQHKIFEPY FTTKS----AGTGLGLSTSYSILKKHKGLITFSSKPGEGTIFYIYLPTTQ
QEE16756.1
Resp reg
PAS1
PAS2
PAS3
Group B
LFAHDINNILQNVLSSVDTILlanEKPEGKEISDDMLKIITEQVNRGAKLVTNVRRLSEI DD--TKIILRDIESFDLLEKAVKYIEESYKDKNLKITIDSE-NRSYNVYANELLLDVFEN ILINAVKYNDNTEIEIIIKITKEkkdnRNFAKFEFMDNGIGIQDERKQIVFERTNRI--- -SFDGMGLGLSLVNHIINKFEGFIWVEDRVKNdytkGSNFAILIPEVT
KKK46579.1
PAS
PAS1
PAS2
NDRKYLNIID---AIVLQLDRKGKILYINVKGNKILGYRENEILGKNWFKTCIPHSKREE MERVFNKVINGE
SEEKYRTLFESSKDGILFTNIEGKIMDCNQTYLDMLGYTLAEI-----------------
PAS1
PAS2
RVFVLDDDKNIHKFLIRAFSHLGLEMVSCYNGDHILEDFQAAEksgkkIDIVFMDLTIPG gIGGKDAVKILRKKYPDLKIIVFSGYSNDPVIANFKDYGFDDFLEKPFSIEQLKAILSKW I
ELRLHSEIMNNISEGVYLIRLENlIIVYANPRFEEMFGYEPNEMIGKYVvIVNAPTDKTP EEIKDEIMGILKET
ETKRLASIVGTSSDSIVSMDKEGMIKSWNKGAERIYGYALEEVLEKS-ISMLIPPDRMEE IPNLIEKVVQGE
RISHELNTPLISILNGSQYLLdfKNNKMSDDVSNIVKIIYQGGYRLREMVNNLITAYELE TEQLIFKIKRENLIPIIKKCVEDLIFQAEKRKIFINLELP-TDLNVDVDKEMIKKAILNL LSNAVKNTLP-NGNIFIKSIEHHNFIDIIIRDTGVGLTDKEIPILFKKFGKIERygkgmD VDIEGPGLGLYIANEIVKLHIGEILVKSKGRNkGSTFILRLFLK-
SMSHELRTPLNSIIGFTDVILerISGEINEEQDKYLTHVKTSAMLLLDLINDILDITKIE SGKMELNIEEVNLSQIISQVDTMIKPIYMKKSLIFEVQEIDKEKVIRVDRLRFKEILFNL LSNAVKYTKE--GKIMIKFSEEEEFWKFDVIDTGIGIKKENFDILFKDFRRIASkyVVAT EGTGLGLSLSKRLVEFHGGTISFTSTFGKGSKFTFTIPKQL
QEE16613.1
KKIRFQRVFDRLPIPIAISDQNDQNLYVNPKFTEILGYTVNDIPNTESWMNLAYPD
KKK43125.1
DSQWYITTLKSIGDAVIATDKEGKILFLNSVAESLTGWSGNDAVGLPLekVFNIINEDTL EKVENPVSTVIKTG
SQQRFRKIVEYFPYPIVVTTPRNDVVYVNPAFTKSLGFTIEDIPNAKTwyLKAYPNPEYR NSIIE
AEIKFQEIIENSRDMILLLNEKGIIQMINPAVTAIMGYSEDDIINHSP-LEFVLPE
LFAHDINNILQNVLSSVDTILlanEKPEGKDISDDMLKTITEQVNRGAKLVSNVRSLSEI DDA--KIVLGEIESFNFIEKAVKYVEECYKEKNLKITIDAE-NRSYNVIANELILDVFEN VLINAVKYNDNTEIEIIIKISKEkkdkRNFTKFEFMDNGIGIQEERKQNVFERTNR---- KSFNGMGLGLSLVNHIISKYEGHIWVEDRVKNdytkGSNFVILIPEVT
KKK45609.1
KKNQLANIVENSNDGIISAELDGTIRSWNKSAEVIFGFSSEEIIGKK--LDLIIPSNRFN EVKYIIDKIKQG
PAS
GLAHDFNNILVSIIGNINLFQmDEDTLTTEQQDMLMDLEKAAMRARDLTNQLLTFSK--- -GGTPIKKPESIEQVIRDSANFILRGSK---SKCTFYFESNLPPVEIDVGQINQVLNNLL INASQAMPHG-GIIDISVFREkidnkssiplipGCYIKLIIEDKGVGIPKSIQHKIFEPY FTTKS----AGTGLGLSTSYSILKKHKGLITFSSKPGEGTIFYIYLPTTQ
KIIIIDDDDDVCDSLSLFLNKVGYKTETAQTGQEGINKITKEFYIVALLDIKLPDIEGTE LLKRLKEKHPKMDVIIMTGNASLNSAVQALNNGASGYILKPINLENLLTNLKNSI
WP_147663689.1
Resp reg
PAS1
PAS2
PAS3
GIAHDFNNILAVILGNVSLLQmDADHLSEEHNLIINDISTASERAKSIIKQLQEFSSQD- -IRKEKAEIFDLSVIVDEVINFMKNTT-NRLIQKENSIPKNRYFIKGNPVEFNQVLLNLG INSIHAIEEKkNGATTLDFIKIdisfpegeqkNTHIVVNFMDSGRGIPEKNLKQMFIPFF TTKTgsgASKIKGRGLGLSMVYHIITNKFdGSINIESRVDHGTVFYITLPLID
Resp. reg.
PAS1
PAS2
PAS3
PAS4
WP_147661239.1
SEEKYRELYENAPNAYFSVNPDKSIKGCNQPACELLGYNKKEILTMK-VFDLYYNSSEGL QKAKrlFKQFLEGK
KNNRLAAIVDSSDDAIIGKRLDGIITSWNTGATKIYGYMAKEVIGKP-INIMVPPDRQDE VTQILEKIKRRE
DSQWYFTTLKSIGDAVIATDKEGKILFLNSVAESLTGWLSNEAVGLPLekVFNIINEETL EKVENPVSTVIKTG
PAS
RVFVLDDDKNIHKFLIRAFSHLGLEMVSCYNGDHILEDFQAAEksgkkIDIVFMDLTIPG gIGGKDAVKILRKKYPDLKIIVFSGYSNDPVIANFKDYGFDDFLEKPFSIEQLKAILSKW I
SEAKFRALFNNTNDSIFIHDGSSQFIEINKTACERLGYTREELLRLS-LKDIIPPGYKID LQTNIKTLYEKG
PAS1
PAS2
PAS3
PAS4
LFFHDINNILQHILSATDLYEnylKLPNDKQKIENIPNIIRNQVLRGAKLISNIRKISDL EIE--KAPIKTVDLCEHLNESVNLLTNSYKDKDIKIVIDTP-LKDLRIEASDLFHDVCDN ILFNAIKHNNKPTPEILIKISKLqkdeRNYIRTEFLDNGRGIEDNRKKQIFQRGFNDDRS T--SGMGLGLSLVKKIIDSYDGDIWVEDKVIGdytkGSKFIVLIPEGI
FGYSQEDFLNHKTvYSDIVHPEDLP KIRSESQHHVKNQ
KKK46537.1
SEEKVQNMITNISDVLLESEPNGILTYISPQIKNIIEYQSEELIGLN-FMDFVHIEDINS FKETASKALKTQ
SEERFRIVAENGSDLIYEFTSGGKKIAWFGDVENFYGLKSDEIPKNFDeVISRLHPSDQR KITKQIEDLSAST
KKIRFQRVFDRLPIPIAISDQNDQNLYVNPKFTEILGYTVNDIPNTESWMNLAYPD
RVSHELQTPIASMNLALQMLLenYKEQLSNNVIDLIEIIQRGERRLRSLIDNVIDASKVE LGKIKLYVQKENLIEIINDCIDVIYFMADKRQISISKDLPE-SLHLNFDKLRIEQVILNL LSNAIKYTPSS-GNIYINIKESEQFADLFIKDTGIGLTEEEQGKIFKKFGKIERedcnsD IRNDGTGLGLYISNELVKLHDFSLRVDSEGRNkGSTFILRIPKN-
SESRFRMVAENTSDLIYEYDYNDKNVKTFGGLESLLGISEKELNKSPKkFYDFIHDEDRL IIEKEMVKFLASK
KKK40441.1
SQQRFRKIVEYFPYPIVVTTPRNDVVYVNPAFTKSLGFTIEDIPNAKTwyLKAYPNPEYR NSIIE
SKERFRTVAQNVSDIIFEYDVKKHSVYWFGDIAEHLKIGDDEIPKRMTaFMKFIHPDDIE RIKSEviiTRESRKNA
AEIKFQEIIENSRDMILLLNEKGIIQMINPAVTAIMGYSEDDIINHSP-LEFVLPE
DVNNIFQNILSSIEISKEYLDDYLEKKEILSDMNNLINDQIVKGKKLVLNVQNLSLVED- -TGLVLSPIEVLDNLEKIIDVIKEDFQHRNIEIQIESE-QSMYIVNADKLLKNIFENIFT NSIKHNQNSNIEIFIRVSRKkkngVNFIKITFIDNGLGIPDLMKLTIFSRDFRK--SGNY TGIGLGLLLVKRILEKYNGEIIVKDRVKGdytkGSKFLILIPEFN
KKK40347.1
Group D
GLAHDFNNILVGILGNVNLIQhSEENFSSEIGEMLEDISEATKKATALTRQLLTFSK--- -GGEPIKKAQDIEKIIEFSMKLVMPGS----KSICKFLPHdKVPPVNVDAGQIEQVINNI LLNADQAMEHG-GIITIALSQIqfkeldrkvmlkpGQYIKISISDQGIGIPPQVQKKIFS PYFSTKP----NGNGLGLATVYSIIKKHNGKISFTSTVDVGTTFDIFLPVSQ
WP_147664078.1
Resp reg
PAS
RTSHELKTPLVSIKGFSNLLL-ELYSDKLDDYVLNTIGEikqGCIRLEDLIRDILKSAEL DSESVQLKKTSEDLSFLIRVCVNELKEFVKLRNHSINLQLQ-DDLITCFEKEQIHQVVSN LLTNAIKFTPI-NGVIELKSIKKEDSLIFSIKDNGIGFIDEEKERLFKQFGKIERYGQGl divsDGSGLGLYISKKIIELHGGKIWLESEGRSkGSTFNFTLPLIS
RVSHELKTPLVSICGGSELLLelFGSDIKEEARELAEIIGKGGTRLKNLVDNLVDVTRIE YNKFELEIELINISDVLQDCAKEMMYLIKKREINFQLDLP-DTLIVNIDRVRIEQVVLNI LSNAIKNTPP-NGKVVMKLTKKKEWVEFLVNDSGIGLTREEMDRIFTRFGKIERYGPGle yidiQGSGLGLFISKEIIDLHKGHIWAESGGRDkGSTFTIKLPIS-
KKK46295.1
KKK46428.1
VASHDLQEPLRMVISFTQLLAkrYNDKLDEDAKDYINFAVDGARRMQTLINDLLSYSRIT TR--GDPFKIVNLKNVLEDIILNLQIKIKETSAKITYGEM---PSLFADKTQLMQLFQNL ISNALRFRSKELPLIHISAQHSYKEWIFSVKDNGIGIEQRFFKKIFVIFQRLHTRDEYPG SGIGLAICKRIVEKLNGRIWVESEVGKGSTFKFAIPLKK
KKK40193.1
KILVLEDDSTVIKLLKKMFHILNQKADFVTDGYNILQLYEQALqnndpYDIVIMDLTIPG gMGGKDTIEALVKIDPDVTAIVSSGYSTDPVMANFSEYNFKGVLEKPFTINEVKKILLRW L
STTLEQAILDAMSEAVFLIDLDNKILQCNKSTVKFLGKSKKDEIIGQTYWELLH
PAS1
PAS2
PAS3
PAS4
PAS1
PAS2
PAS3
PAS4
SKGRYQRLIKSVKEGYYEVDLKGNFTFVNKALSRFIGYSQEEMIGQS-YKKITDRTKRKL VFKTFNKVFKTE
ISNKFETVIENLIDMIAILDFKGEFLYVSPQVYNITGFTQEEIIGKNG-FTFIHPNDLDK VLKTMARLLKER
SEDKYSNLFQHSNDGIFLHDLDGNITDVNRKVLEQLGYTKSEILSLK--VSQLHPTSEMV ESKKaFDKISENG
SEEKYKNLVEFSTDFIFSEDLHGEFIYINPTFNKTLGYSNEELLHKD-ISHLIHPEDLEG LIKAKLPLLEGK
DGVFRYFNKQFEEIIGFSREEINGWSSgeFLEAIHPDDR DFVREQARKKQQGA
Group C
SEEKYRSMVNNLDLGFYQVDWDGNLLNYNPAFASLFGFEPDEDITNVNVKEF--------
GIAHDFNNILTAVLGNTALLKMDLDQNSEKFTILNELESAIHRASKLTHQLLTFSK---- ----GGSPIKKFMFIGDVIKESANFALhGTKNEIEFNFAPGLWPVHIDEGQISQVINNLV INAVQATNI-NSKIIIQANNYlmepennmmltpGNYVKISFQDFGNGILEENQEKIFDPY FTTKE----TGSGLGLSVCYSILTRHSGKIKVTSKIGEGSTFTFYLPAHP
DDKVEYVNSTVLQIFEYSFEEVKNWSMkdLINIIHPDDL KLLRKSrLEMRTNNS
WP_147662496.1
Resp reg
PAS1
PAS2
SMSHELRTPLNSIIGFSDVLLekYYGELNDQQEIYLNNVKSSAGHLLNLINDILDISKIE AGKVELNIQKISIHKVLKQTEITLRPEFEKKNLKFEMVGLNSEQVVPVDLIRFKEILYNL LSNAIKYTKK--GSVKLEVIEEEDLWKFNVIDTGIGIKEEHFDLIFNEFERIQSeyVSSV EGTGLGLSLTKRLVELHGGNISFTSKWEEGSIFTFTLPKK-
SESKYRNLIESVPFSIALINLGGRVIYCNPATEKLLGFKEEELVGYEFrNLPIINQKYLP MMLKRFKKAANGE
WP_147663673.1
RTSHELKTPLVSIKGYADLLL-TQHYEELDFYTISILYEmkrGCSRLESLIKDLLESSEL ESEQIKLNKTEEDLTFLIRFCIKDLQGLVENRSHNLILEIQ-DRLKATFEKEKIYEVITN LLSNAIKYTPPS-GEIRIKSEIKDNDIVISIQDNGIGLTREETLKLFKKFGKIERygqgl DVDSEGSGLGLYISKKIIELHGGNIWVESEGRNlGTTFFFSLPIIK
RVSHELKTPLVSICGGSELLLelFGSDIKEEARELAEMIGKGGTRLKNLVDNLLDVTRIE YNKFELEKESINISEVLQDCAEEMMYLTKKREINFQLDLP-DTLIVNIDRVRIEQVVLNI LTNAIKNTPP-NGKVFMKLTKKKEWVEFSVNDSGIGLTREEMDRIFTRFGKIERYGPGle yidiQGSGLGLFISKEIIDLHKGQIWAESGGRDkGSTFTIKLPIS-
KKK46292.1
KKK44457.1
RILILEDEKPIRVLLEKFLRKKNFYVESAADGEEILIKYQDALdkgqeFDILILDLTIkG GMGGEETFQKLLEINPNVKAIVASGYSTKKILSDYKQYGFAGMLSKPFKMSDLLKEIERL L
PAS1
PAS2
INAKLQSVFKTSNEGFWESNNEDITIDVNPKMCEILGRTREEIIGRDS-HELLTPDGKDK ISFQHEEYRKKG
DNYILANIVENSNDAIISLNIDGSIISWNKGAENVFGYKEKEIKGKN--YSLLGPENLTI DQTLIFNEVKNS
KTLFFQKILNNLPEPAFAVDSNCKIIEWNKAMEEFSGIKAELIIGKT
PAS1
PAS2
RASHELKTPLISIKGYSDLILslYTDQLGSEIISYLNEISHGCERLQYIINDLLKSSRLE SIKLKSNLQKEDLPFLIKYCVNGMESLAKKRKQSIELNIH-DQLYVNIEKEEIHAVLSNL LSNAIKYTPP-NGKIEVKTDLKDDLVVISVTDNGIGFTEEQRKIVFKQFGKIERfgqglD LEIDGTGLGLYISKRIVESHGGKIWMESEGKNmGASFYFTLPTVK
SEEKYRSMVNNLDLGFYQVDWDGNLLNYNPAFASLFGFEPDE------------------
KKK43837.1
SMSHELRTPLNSIIGFSDVLLekYYGELNDQQEIYLNNVKSSAGHLLNLINDILDISKIE AGKVELNIQKISIHKVLKQTEITLRPEFEKKNLKFEMVGLNSEQVVPVDLIRFKEILYNL LSNAIKYTKK--GSVKLEVIEEEDLWKFNVIDTGIGIKEEHFDLIFNEFERIQSeyVSSV EGTGLGLSLTKRLVELHGGNISFTSKWEEGSIFTFTLPKK-
EKEMLAVTLAGIVDGVITLDADANIILINKVAENILEMDYDEVFGKKIfnITTLYKDKSR DPYELYeVKNMLGKR
QEE16740.1
SESRYRNLIESVPFSIALIDQGGKVIYCNPATEKLLGYKREELIGNEFrSLPVINQKYLP MMLNGFKKVANGE
GIAHDFNNLLQAVFGHLDLILlEFENNNDKDKEILDSLFKiksAAEHGAEITHQLLTLSK IHN----VEFSSININDEILEIYSILKHSIP-KMVEINLELSEDMDPIYGNSTSIQQVIM NLVLNARDALQDG-GKISIKTDEVilddlfcemfanltpGKYISITITDTGIGMSQEVLE HLFEPYYSTKP--HGKGTGLGLSIVYNIIQQHNGSIQCYSEVDRGTRFKIFLPVNE
WP_147664519.1
Resp reg
PAS
RVSHELKTPMTSIYGSIQILLtiYNKDLSEEVLNYVKIGHRGCLRLKQLIENLLDASRLE SKKFELSLLKENLVEIIVDCVEDLNYLVHNRHLLITLDLP-DEIQADIDSLRFRQVITNI ISNAIKNTPK-EGEIFIHIIEDADKIDILIRDTGVGLTKQEQEKLFGKFGKIERygmdlD VDIEGCGLGLYISKEIIELHKGQILVESEGRNkGSTFTIRLFK--
KKK46009.1
LYVNQQFAKIFGYSVEEILNWEFggYLKTVHLEDL EFVKNQaekkqkgIENVINRY
INAKLQSVFKTSNEGFWESNNEDITIDVNPKMCEILGRTREEIIGRDS-HELLTPDGKDK ISFQHEEYRKKG
PAS1
PAS2
PAS1
PAS2
DNYILANIVENSNDAIISLNIDGSIISWNKGAENVFGYKEKEIKGKN--YSLLGPENLTI DQTLIFNEVKNS
SEEKFRTIAEQSSAGFIIIQNSQIIYLNNAVSKVFGYSSEELKNISIidIFKFVHPEDR
IKIDYADLFEYSLDFLFILDLKGNIIDINNVATETLGYSRREILNMN-ITEFLLEEEIEQ LLNKRKELINIG
TILVIDDNEEILEYTIKILKKFGYNVLIANSCKNIHEILKRNKrIDLFIVDYIMPHTGGI KCIRNIReKLDYNTKYILSSGMGSEKLNEECEINKIDTFLPKPYTMGDLLLIIQSVL
SMSHELRTPLNSIIGFSDILKekFYGDLNEKQENYIENVRSSAQHLLELINDILDISKIE AGKITLRFVDVNLKELLNQIGTTIRPEYEKKNLTFEIIGLEEGKMIKCDPKRFKEIIFNL LSNAIKYTIS--GGITLEIFETRNHWEFSVIDTGIGIEEKDFKFIFQDFKRLKNehTATV EGTGLGLSLTKRLIELHNGNISFKSKWKEGSTFTITLPKFS
PAS1
PAS2
PAS3
PAS4
QEE16628.1
RASHELKTPLISIKGFSELILssyKEELNPEVISNLTEIIKG-CRRLQTIINDLLHASRL ETIDPKPRLEEEDLTFLIKYCVNELKSVIVKRRHSVNLEVH-DSLIARFEKEEIHDVISN LLINAIKYTPP-NGKINIKMELLEDTVIISVSDNGIGFNQYQKNSIFKQFGKIERYGQGl dlgiDGTGLGLYISKRIVESHGGKIWMESEGINqGSTFYFSIPL--
SEEKFRTLFEKANDGILLSDAETwRIFTGNQKICQMLGYSLEEI
KKK44257.1
DGVFKYVNDVVSEIIGYDIQDILNWSQneFFNKIHPSDV PIALKRFQQMQLGE
SQGRFQELVETMDEGIYEYDLSGNCTYSNEAHYKVLGYHVNE
SEEKFRTIAEQSFMGIIIVQEGKLKYLNKALAKMLEYPFEEMIKWSDkeMAEIVHPDDLEYIQKRLQSNREGT
PAS1
PAS2
PAS3
GIAHDFNNVLTVILGLSNLTIdslRDENEEINRNELIKNLEDvlrAGDRAAKLTKDVLTF SRKQ----VFQSQIVNLNKLLKNNKDMLARVL-GENISFNLKLDQENKSIEIDPNQMSLV ILNMAINSRDAMPNG-GDFTIETSRTylskglkgfnlkpGNYIKISLRDTGIGMDETHKQ HLFEPFFTTKE--KGKGTGLGLSIAFGIIKQYHGEIKVESKLGTGTVFHIYLPEID
QEE16389.1
Resp reg
SSKKYSNLVDNLTDIVGVIDKKGKIIYGNSQMYTSLGYKPEELKGQNFFALYLDPK----
RISHEIKTPLTSIYTTTNLLLnvYKEEFSNKILDFIKMINEGGERLKELVDNMLDVYDIE SNMIKLNFKAHNLVDILEMSITTLQPQIVKRQHKVEINLP-HEVYMDVDYDRIKQVFLNL LINALKYTPPG-GKIFVQLQENNSHIDITIKDFGVGFTEQEKGKLFMKFGKIERygkgmN VDTEGAGFGLYIAKELTRLHNGELILKSEGRNkGSTFIIRLFKEI
KKK45741.1
SGEKYRRLFESSTDGIVSTDMEGRLIDFNKAFLEMLGYSKEELL
SLKKYTNLVNNLTDIIGAVDPKGKINYCNPQVYTLLGYKPEELLGQKFFKLLANPK----
PAS
EKENLLKMVENSDDAFINMDLDGTMIYWNKGAEQTFGYTAKEVLYRKN-LDLVPPDRYEE RKFIFDRIKKGE
TILLVEDEEMVRKFAFRVLNSHGYNCLEAKSPKEAIKISQEYHekIDLLLSDVIMPDFSG LELKKKIEKRHPDMIILFISGYDANIISNNGELEVGINLLKKPFSSIELLERIHLLL
SEEKFRTIAE-QSVMGILIVQDGVIKYANESLSEINEYSIQEMMNWSPseLTKAIHPDER GIINERIEKISLGS
PAS

## Slide 2
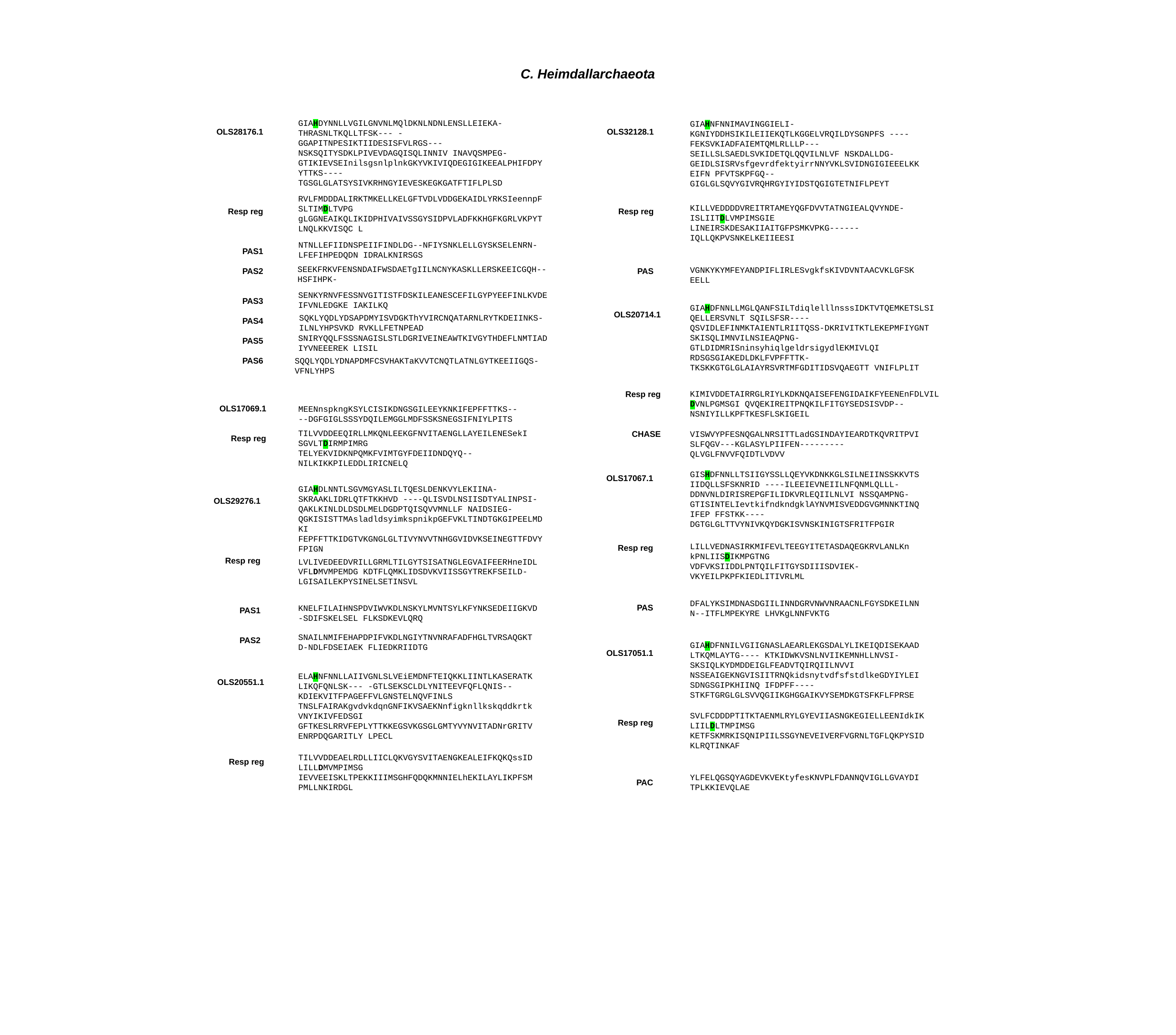

C. Heimdallarchaeota
GIAHNFNNIMAVINGGIELI-KGNIYDDHSIKILEIIEKQTLKGGELVRQILDYSGNPFS ----FEKSVKIADFAIEMTQMLRLLLP---SEILLSLSAEDLSVKIDETQLQQVILNLVF NSKDALLDG-GEIDLSISRVsfgevrdfektyirrNNYVKLSVIDNGIGIEEELKKEIFN PFVTSKPFGQ--GIGLGLSQVYGIVRQHRGYIYIDSTQGIGTETNIFLPEYT
GIAHDYNNLLVGILGNVNLMQlDKNLNDNLENSLLEIEKA-THRASNLTKQLLTFSK--- -GGAPITNPESIKTIIDESISFVLRGS---NSKSQITYSDKLPIVEVDAGQISQLINNIV INAVQSMPEG-GTIKIEVSEInilsgsnlplnkGKYVKIVIQDEGIGIKEEALPHIFDPY YTTKS----TGSGLGLATSYSIVKRHNGYIEVESKEGKGATFTIFLPLSD
OLS32128.1
Resp reg
PAS
OLS28176.1
Resp reg
PAS1
PAS2
PAS3
PAS4
PAS5
PAS6
RVLFMDDDALIRKTMKELLKELGFTVDLVDDGEKAIDLYRKSIeennpFSLTIMDLTVPG gLGGNEAIKQLIKIDPHIVAIVSSGYSIDPVLADFKKHGFKGRLVKPYTLNQLKKVISQC L
KILLVEDDDDVREITRTAMEYQGFDVVTATNGIEALQVYNDE-ISLIITDLVMPIMSGIE LINEIRSKDESAKIIAITGFPSMKVPKG------IQLLQKPVSNKELKEIIEESI
NTNLLEFIIDNSPEIIFINDLDG--NFIYSNKLELLGYSKSELENRN-LFEFIHPEDQDN IDRALKNIRSGS
SEEKFRKVFENSNDAIFWSDAETgIILNCNYKASKLLERSKEEICGQH--HSFIHPK-
VGNKYKYMFEYANDPIFLIRLESvgkfsKIVDVNTAACVKLGFSKEELL
SENKYRNVFESSNVGITISTFDSKILEANESCEFILGYPYEEFINLKVDEIFVNLEDGKE IAKILKQ
GIAHDFNNLLMGLQANFSILTdiqlelllnsssIDKTVTQEMKETSLSIQELLERSVNLT SQILSFSR----QSVIDLEFINMKTAIENTLRIITQSS-DKRIVITKTLEKEPMFIYGNT SKISQLIMNVILNSIEAQPNG-GTLDIDMRISninsyhiqlgeldrsigydlEKMIVLQI RDSGSGIAKEDLDKLFVPFFTTK-TKSKKGTGLGLAIAYRSVRTMFGDITIDSVQAEGTT VNIFLPLIT
OLS20714.1
Resp reg
CHASE
SQKLYQDLYDSAPDMYISVDGKThYVIRCNQATARNLRYTKDEIINKS-ILNLYHPSVKD RVKLLFETNPEAD
SNIRYQQLFSSSNAGISLSTLDGRIVEINEAWTKIVGYTHDEFLNMTIADIYVNEEEREK LISIL
SQQLYQDLYDNAPDMFCSVHAKTaKVVTCNQTLATNLGYTKEEIIGQS-VFNLYHPS
KIMIVDDETAIRRGLRIYLKDKNQAISEFENGIDAIKFYEENEnFDLVILDVNLPGMSGI QVQEKIREITPNQKILFITGYSEDSISVDP--NSNIYILLKPFTKESFLSKIGEIL
MEENnspkngKSYLCISIKDNGSGILEEYKNKIFEPFFTTKS-- --DGFGIGLSSSYDQILEMGGLMDFSSKSNEGSIFNIYLPITS
OLS17069.1
Resp reg
TILVVDDEEQIRLLMKQNLEEKGFNVITAENGLLAYEILENESekISGVLTDIRMPIMRG TELYEKVIDKNPQMKFVIMTGYFDEIIDNDQYQ--NILKIKKPILEDDLIRICNELQ
VISWVYPFESNQGALNRSITTLadGSINDAYIEARDTKQVRITPVI SLFQGV---KGLASYLPIIFEN---------QLVGLFNVVFQIDTLVDVV
GISHDFNNLLTSIIGYSSLLQEYVKDNKKGLSILNEIINSSKKVTSIIDQLLSFSKNRID ----ILEEIEVNEIILNFQNMLQLLL-DDNVNLDIRISREPGFILIDKVRLEQIILNLVI NSSQAMPNG-GTISINTELIevtkifndkndgklAYNVMISVEDDGVGMNNKTINQIFEP FFSTKK----DGTGLGLTTVYNIVKQYDGKISVNSKINIGTSFRITFPGIR
OLS17067.1
Resp reg
PAS
GIAHDLNNTLSGVMGYASLILTQESLDENKVYLEKIINA-SKRAAKLIDRLQTFTKKHVD ----QLISVDLNSIISDTYALINPSI-QAKLKINLDLDSDLMELDGDPTQISQVVMNLLF NAIDSIEG-QGKISISTTMAsladldsyimkspnikpGEFVKLTINDTGKGIPEELMDKI FEPFFTTKIDGTVKGNGLGLTIVYNVVTNHGGVIDVKSEINEGTTFDVYFPIGN
OLS29276.1
Resp reg
PAS1
PAS2
LILLVEDNASIRKMIFEVLTEEGYITETASDAQEGKRVLANLKnkPNLIISDIKMPGTNG VDFVKSIIDDLPNTQILFITGYSDIIISDVIEK-VKYEILPKPFKIEDLITIVRLML
LVLIVEDEEDVRILLGRMLTILGYTSISATNGLEGVAIFEERHneIDLVFLDMVMPEMDG KDTFLQMKLIDSDVKVIISSGYTREKFSEILD-LGISAILEKPYSINELSETINSVL
DFALYKSIMDNASDGIILINNDGRVNWVNRAACNLFGYSDKEILNNN--ITFLMPEKYRE LHVKgLNNFVKTG
KNELFILAIHNSPDVIWVKDLNSKYLMVNTSYLKFYNKSEDEIIGKVD-SDIFSKELSEL FLKSDKEVLQRQ
SNAILNMIFEHAPDPIFVKDLNGIYTNVNRAFADFHGLTVRSAQGKTD-NDLFDSEIAEK FLIEDKRIIDTG
GIAHDFNNILVGIIGNASLAEARLEKGSDALYLIKEIQDISEKAADLTKQMLAYTG---- KTKIDWKVSNLNVIIKEMNHLLNVSI-SKSIQLKYDMDDEIGLFEADVTQIRQIILNVVI NSSEAIGEKNGVISIITRNQkidsnytvdfsfstdlkeGDYIYLEISDNGSGIPKHIINQ IFDPFF----STKFTGRGLGLSVVQGIIKGHGGAIKVYSEMDKGTSFKFLFPRSE
OLS17051.1
Resp reg
PAC
ELAHNFNNLLAIIVGNLSLVEiEMDNFTEIQKKLIINTLKASERATKLIKQFQNLSK--- -GTLSEKSCLDLYNITEEVFQFLQNIS--KDIEKVITFPAGEFFVLGNSTELNQVFINLS TNSLFAIRAKgvdvkdqnGNFIKVSAEKNnfigknllkskqddkrtkVNYIKIVFEDSGI GFTKESLRRVFEPLYTTKKEGSVKGSGLGMTYVYNVITADNrGRITVENRPDQGARITLY LPECL
OLS20551.1
Resp reg
SVLFCDDDPTITKTAENMLRYLGYEVIIASNGKEGIELLEENIdkIKLIILDLTMPIMSG KETFSKMRKISQNIPIILSSGYNEVEIVERFVGRNLTGFLQKPYSIDKLRQTINKAF
TILVVDDEAELRDLLIICLQKVGYSVITAENGKEALEIFKQKQssIDLILLDMVMPIMSG IEVVEEISKLTPEKKIIIMSGHFQDQKMNNIELhEKILAYLIKPFSMPMLLNKIRDGL
YLFELQGSQYAGDEVKVEKtyfesKNVPLFDANNQVIGLLGVAYDITPLKKIEVQLAE
